# Supplementary material for: Tailoring oral targeted therapies dosage in lung cancer: A systematic review of pharmacokinetics studies on renal and hepatic impairment
Source: PLoS One. 2025 Jul 29;20(7):e0324056. doi: 10.1371/journal.pone.0324056 (PMC12306784; doi:10.1371/journal.pone.0324056)
Supplement: S2 File — (DOCX) [file pone.0324056.s002.docx]

Supplementary Material: Tailoring Targeted Therapies Dosage in Lung Cancer: A Systematic Review of Pharmacokinetics Studies on Renal and Hepatic Impairment

Contents

[Supplementary Table 1. Search strategy (performed on May 1, 2024) 2](#_Toc201950058)

[Supplementary Table 2. Search result for each drug. 3](#_Toc201950059)

[Supplementary Table 3. Study Characteristics 4](#_Toc201950060)

[Supplementary Table 4. Risk of bias in case reports and case series 7](#_Toc201950061)

[Supplementary Table 5. Risk of bias in pharmacokinetic (PK) studies 8](#_Toc201950062)

[Supplementary Table 6. Risk of bias in population pharmacokinetic (PopPK) studies and physiologically based pharmacokinetic (PBPK) modelling 9](#_Toc201950063)

[Supplementary Table 7. Drug dosing in hepatic and renal impairment based on drug label and pharmacokinetic studies on impairment of renal and hepatic function. 10](#_Toc201950064)

[Table S8. Dose adjustment for oral targeted therapy in hepatic impairment population 25](#_Toc201950065)

[Table S9. Dose adjustment for oral targeted therapy in renal impairment population 26](#_Toc201950066)

[Table S10. Dose adjustment for oral targeted therapy in HD/CAPD population 28](#_Toc201950067)

[References 30](#_Toc201950068)

# Supplementary Table 1. Search strategy (performed on May 1, 2024)

| **Database** | **Keywords** | **Hits** |
| --- | --- | --- |
| Pubmed | ((Afatinib[Title/Abstract] OR Erlotinib[Title/Abstract] OR Dacomitinib[Title/Abstract] OR Osimertinib[Title/Abstract] OR Gefitinib[Title/Abstract] OR Alectinib[Title/Abstract] OR Brigatinib[Title/Abstract] OR Ceritinib[Title/Abstract] OR Crizotinib[Title/Abstract] OR Lorlatinib[Title/Abstract] OR Dabrafenib[Title/Abstract] OR Trametinib[Title/Abstract] OR Vemurafenib[Title/Abstract] OR Larotrectinib[Title/Abstract] OR Entrectinib[Title/Abstract] OR Capmatinib[Title/Abstract] OR Tepotinib[Title/Abstract] OR Selpercatinib[Title/Abstract] OR Pralsetinib[Title/Abstract] OR Cabozantinib[Title/Abstract] OR Sotorasib[Title/Abstract]) AND ((((hepatic[Title/Abstract]) OR (renal[Title/Abstract])) OR (hemodialysis[Title/Abstract])) OR (peritoneal dialysis[Title/Abstract]))) AND ((pharmacokinetic[Title/Abstract]) OR (pharmacokinetics[Title/Abstract])) | 131 |
| Scopus | ( TITLE-ABS-KEY ( afatinib OR erlotinib OR dacomitinib OR osimertinib OR gefitinib OR alectinib OR brigatinib OR ceritinib OR crizotinib OR lorlatinib OR dabrafenib OR trametinib OR vemurafenib OR larotrectinib OR entrectinib OR capmatinib OR tepotinib OR selpercatinib OR pralsetinib OR cabozantinib OR sotorasib ) ) AND ( TITLE-ABS-KEY ( hepatic ) OR TITLE-ABS-KEY ( renal ) OR TITLE-ABS-KEY ( hemodialysis ) OR TITLE-ABS-KEY ( peritoneal AND dialysis ) ) AND ( TITLE-ABS-KEY ( pharmacokinetic ) OR TITLE-ABS-KEY ( pharmacokinetics ) ) AND ( LIMIT-TO ( DOCTYPE , "ar" ) ) | 253 |
| Web of Science | (AB=(afatinib OR erlotinib OR dacomitinib OR osimertinib OR gefitinib OR alectinib OR brigatinib OR ceritinib OR crizotinib OR lorlatinib OR dabrafenib OR trametinib OR vemurafenib OR larotrectinib OR entrectinib OR capmatinib OR tepotinib OR selpercatinib OR pralsetinib OR cabozantinib OR sotorasib)) AND (((AB=(hepatic) OR AB=(renal)) OR AB=(hemodialysis) OR AB=(peritoneal dialysis)) AND (AB=(pharmacokinetic) OR AB=(pharmacokinetics)) | 118 |

# Supplementary Table 2. Search result for each drug.

| **Drug Name** | **Pubmed (Medline)** | **Scopus** | **Web of Science** |
| --- | --- | --- | --- |
| **Targeted therapy** | | | |
| **EGFR Inhibitors** | | | |
| Afatinib | 9 | 20 | 9 |
| Erlotinib | 19 | 43 | 17 |
| Dacomitinib | 3 | 4 | 3 |
| Osimertinib | 5 | 12 | 4 |
| Gefitinib | 18 | 36 | 17 |
| **ALK Inhibitors** | | | |
| Alectinib | 6 | 14 | 6 |
| Brigatinib | 4 | 7 | 4 |
| Ceritinib | 3 | 11 | 3 |
| Crizotinib | 14 | 28 | 12 |
| Lorlatinib | 2 | 5 | 1 |
| **BRAF/MEK Inhibitors** | | | |
| Dabrafenib | 3 | 6 | 3 |
| Trametinib | 5 | 7 | 5 |
| Vemurafenib | 5 | 8 | 4 |
| **NTRK Inhibitors** | | | |
| Larotrectinib | 2 | 4 | 2 |
| Entrectinib | 3 | 6 | 3 |
| **MET Inhibitors** | | | |
| Capmatinib | 2 | 5 | 2 |
| Tepotinib | 1 | 2 | 1 |
| **RET Inhibitors** | | | |
| Selpercatinib | 0 | 1 | 0 |
| Pralsetinib | 1 | 1 | 0 |
| **Multi-targeted Tyrosine Kinase Inhibitors (TKI):** MET, VEGFR, and RET | | | |
| Cabozantinib | 25 | 31 | 21 |
| **KRAS Inhibitor** | | | |
| Sotorasib | 1 | 2 | 1 |
| **Total** | 131 | 253 | 118 |

# Supplementary Table 3. Study Characteristics

| No | Author (year) | Drug studied | Study type | Condition studied | Dose studied | Patients |
| --- | --- | --- | --- | --- | --- | --- |
| 1 | Wiebe (2016) [1] | Afatinib | PK | RI | 40 mg | NSCLC patients   - eGFR 15 – 29 = 8 - eGFR 30 – 59 = 8 - eGFR > 90 = 14 |
| 2 | Schnell (2014) [2] | Afatinib | PK | HI | Initial dose: 50 mg | NSCLC patients   - Child-Pugh A = 8 - Child-Pugh B = 11 - Control = 16 |
| 3 | Freiwald (2014) [3] | Afatinib | PopPK | HI and RI | Initial dose: 20, 40, or 50 mg | 927 solid tumor patients (4460 concentrations); 82,4% NSCLC |
| 4 | Nakao (2019) [4] | Afatinib | PopPK | HI and RI | 40 mg | 34 NSCLC patients (254 concentrations) |
| 5 | Yamaguchi (2015) [5] | Afatinib | Case Report | HD | 30 mg multiple dose | 59 y.o. with NSCLC |
| 6 | Imai (2017) [6] | Afatinib | Case Series | HD | Initial dose: 30 mg | 3 haemodialysis NSCLC patients |
| 7 | Miller (2007) [7] | Erlotinib | PK | HI and RI | Initial dose:  50 mg in HI  75 mg in RI | 55 EGFR positive tumor; 9,3% NSCLC |
| 8 | O’Bryant (2012) [8] | Erlotinib | PK | HI | 150 mg | 36 solid tumor patients; 11,1% NSCLC |
| 9 | Czejka (2013) [9] | Erlotinib | Case Report | HI | 150 mg | 62 y.o. with pancreas adenocarcinoma |
| 10 | Togashi (2010) [10] | Erlotinib | PK | HD | 150 mg | NSCLC patients   - 3 with haemodialysis - 5 with normal organ function |
| 11 | Horak (2010) [11] | Gefitinib | PK | HI | 250 mg single dose  250 mg multiple doses for 28 days | 40 solid tumor with cirrhosis:  10 on each (normal, CP A, CP B, and CP C)  Patient with liver metastasis   - Moderate HI = 16 - Severe HI = 7 - Control = 18 |
| 12 | Shinagawa (2007) [12] | Gefitinib | Case Report | HD | 250 mg multiple dose | 58 y.o. with NSCLC |
| 13 | Luo (2016) [13] | Gefitinib | Case Report | HD | 250 mg multiple dose | 76 y.o. with NSCLC |
| 14 | Yamaguchi (2015) [14] | Gefitinib | Case Report | CAPD | 250 mg multiple dose | 72 y.o. with NSCLC |
| 15 | Giri (2015) [15] | Dacomitinib | PK | HI | 30 mg single dose | Phase I study   - Mild HI = 8 - Moderate HI = 9 - Control = 8 |
| 16 | Piscitelli (2022) [16] | Dacomitinib | PK | HI | 30 mg single dose | healthy subject (phase I)   - Severe HI = 8 - Control = 8 |
| 17 | Grande (2019) [17] | Osimertinib | PK | HI | 80 mg single dose | Malignant solid tumor (20% of them is lung cancer)   - CP A: 7 - CP B: 5 - Control: 10 |
| 18 | Vishwanathan (2020) [18] | Osimertinib | PK | RI | 80 mg single dose (part I); 80 mg multiple dose (part II) | Solid, malignant tumor  Part I   - eGFR <30 = 7 - eGFR > 90 = 9   Part II   - eGFR <30 = 3 - eGFR > 90 = 7 |
| 19 | Fujiwara (2023) [19] | Osimertinib | PK | RI | 80 mg in group A and B; 40 mg in group C | A: eGFR > 50 = 12  B: eGFR 30 – 50 = 8  C: eGFR <30 or HD = 2 |
| 20 | Matsunashi (2020) [20] | Osimertinib | Case Report | HD | 80 mg multiple dose | 66 y.o. with NSCLC |
| 21 | Tabata (2023) [21] | Osimertinib | Case Report | HD | 240 to 560 mg per week | 78 y.o with NSCLC |
| 22 | Morcos (2018) [22] | Alectinib | PK | HI | 300 mg single dose | Phase I study   - CP B = 8 - CP C = 8 - Control = 12 |
| 23 | Alsmadi (2021) [23] | Alectinib | PBPK | HI | 300 mg single dose | NA |
| 24 | Park (2022) [24] | Alectinib | Case Report | HD | 300 mg multiple dose | 55 y.o. with NSCLC |
| 25 | Gupta (2021a) [25] | Brigatinib | PopPK | HI and RI | 10-300 mg single or multiple dose | 442 participants (105 healthy volunteers and 337 patients with NSCLC) |
| 26 | Gupta (2021b) [26] | Brigatinib | PK | RI | 90 mg multiple dose | Phase I study   - eGFR >90 = 8 - eGFR <30 = 8 |
| 27 | Hanley (2023) [27] | Brigatinib | PK | HI | 90 mg multiple dose | Phase I study   - CP A = 6 - CP B = 6 - CP C = 6 - Control = 9 |
| 28 | Hong (2017) [28] | Ceritinib | PopPK | HI and RI | 50-750 mg single or multiple dose | 581 patients (NSCLC and non-NSCLC) |
| 29 | Wang (2016) [29] | Crizotinib | PopPK | HI and RI | 250 mg multiple dose | 1214 patients (97% of which is NSCLC) |
| 30 | El-Khoueiry (2018) [30] | Crizotinib | PK | HI | A1: 250 BID A2: 200 QD  B: 250 BID  C1: 250 QD  C2: 200 BID  D: 250 QD | A1 = normal (8)  A2 = normal (9)  B = mild HI (10)  C1 = moderate HI (7)  C2 = moderate HI (8)  D = severe HI (6) |
| 31 | Tan (2017) [31] | Crizotinib | PK and PBPK | RI | 250 mg single dose | - eGFR >90 = 8 - eGFR <30 = 8 |
| 32 | Chen (2021) [32] | Lorlatinib | PopPK | HI and RI | 10-100 mg single and multiple dose | 330 patients NSCLC, 95 healthy participants |
| 33 | Lin (2022) [33] | Lorlatinib | PK | RI | 100 mg single dose | Phase I study   - Normal = 8 - 60-90 = 8 - 30-60 = 8 - <30 = 5 |
| 34 | Oullet (2014) [34] | Dabrafenib | PopPK | HI and RI | 12-300 mg multiple dose | 595 subjects (95.6% of which is melanoma) |
| 35 | Park (2017) [35] | Dabrafenib and trametinib | Case report | HD | 75 mg once daily | 78 y.o. with metastatic melanoma |
| 36 | Oullet (2016) [36] | Trametinib | PopPK | HI and RI | 0.125-10 mg single and multiple dose | 493 subjects (3,120 concentration) |
| 37 | Voon (2022) [37] | Trametinib | PK | HI | 1-2 mg multiple dose | Phase I study   - Mild HI = 6 - Moderate HI = 3 - Severe HI = 3 - Control = 10 |
| 38 | Chen (2022) [38] | Capmatinib | PK | HI | 200 mg single dose | Phase I study   - Mild HI = 6 - Moderate HI = 8 - Severe HI = 6 - Control = 9 |
| 39 | Xiong (2022) [39] | Tepotinib | PopPK | HI and RI | 30-1400 mg single and multiple dose | 613 subjects (10,788 concentrations) |
| 40 | Nguyen (2019) [40] | Cabozantinib | PopPK | HI | 60 or 100 mg multiple dose | 489 hepatocarcinoma patients |
| 41 | Nguyen (2016) [41] | Cabozantinib | PK | HI and RI | 60 mg single dose | Phase I study   - Mild HI = 8 - Moderate HI = 8 - Control = 10 - eGFR 30-60: 10 - eGFR 60-90: 10 - eGFR >90: 10 |
| 42 | Gerner (2021) [42] | Cabozantinib | PBPK | HI | 60 mg single dose | NA |
| 43 | Zimmermann (2021) [43] | Cabozantinib | Case report | HD | 80 mg multiple dose | 34 y.o. with adrenocortical carcinoma |
| 44 | Cheung (2024) [44] | Pralsetinib | PK | HI | 200 mg single dose | Phase I study   - Moderate HI = 8 - Severe HI = 6 - Control = 13 |

BW = body weight; HI = hepatic impairment; RI = renal impairment; PK = pharmacokinetic study; PopPK = population pharmacokinetic study; PBPK = physiologically based pharmacokinetic study

# Supplementary Table 4. Risk of bias in case reports and case series

| First author, year | Selection | Ascertainment | Causality | Reporting | Conclusion |
| --- | --- | --- | --- | --- | --- |
| Yamaguchi (2015) | + | + | + | + | Low Risk |
| Imai (2017) | + | + | + | + | Low Risk |
| Czejka (2013) | + | + | + | + | Low Risk |
| Shinagawa (2007) | + | + | + | + | Low Risk |
| Luo (2016) | + | + | + | + | Low Risk |
| Yamaguchi (2015) | + | + | + | + | Low Risk |
| Matsunashi (2020) | + | + | + | + | Low Risk |
| Tabata (2023) | + | + | + | + | Low Risk |
| Park (2022) | + | + | ? | + | Unclear Risk |
| Park (2017) | + | + | ? | + | Unclear Risk |
| Zimmermann (2021) | + | + | ? | ? | Unclear Risk |

Note: +: low risk of bias; ?: unclear risk of bias; -: high risk of bias

# Supplementary Table 5. Risk of bias in pharmacokinetic (PK) studies

| First author (year) | Selection | | | | Comparability | | Exposure | | | Total |
| --- | --- | --- | --- | --- | --- | --- | --- | --- | --- | --- |
|  | Definition of exposure | Representativeness | Selection of controls | Definition of controls | On age | On other risk factor | Assessment of exposure | Same methods of ascertainment for case and controls | nonresponse rate |  |
| Wiebe (2016) | * | * | * | * | - | * | * | * | * | 7 |
| Schnell (2014) | * | * | * | * | - | * | * | * | * | 7 |
| Miller (2007) | * | * | - | * | - | * | * | * | * | 6 |
| O’Bryant (2012) | * | * | * | * | - | * | * | * | * | 7 |
| Togashi (2010) | * | - | * | * | - | * | * | * | * | 6 |
| Horak (2010) | * | * | * | * | - | * | * | * | * | 7 |
| Giri (2015) | * | * | * | * | - | * | * | * | * | 7 |
| Piscitelli (2022) | * | * | * | * | - | * | * | * | * | 7 |
| Grande (2019) | * | - | * | * | - | * | * | * | * | 6 |
| Vishwanathan (2020) | * | - | * | * | - | * | * | * | * | 6 |
| Fujiwara (2023) | * | - | * | * | - | * | * | * | * | 6 |
| Morcos (2018) | * | * | * | * | - | * | * | * | * | 7 |
| Gupta (2021b) | * | * | * | - | - | * | * | * | * | 7 |
| Hanley (2023) | * | - | - | * | - | * | * | * | * | 6 |
| El-Khoueiry (2018) | * | - | - | * | - | * | * | * | * | 6 |
| Tan (2017) | * | * | * | * | - | * | * | * | * | 8 |
| Lin (2022) | * | - | * | * | - | * | * | * | * | 7 |
| Voon (2022) | * | - | - | * | - | * | * | * | * | 6 |
| Chen (2022) | * | * | * | * | - | * | * | * | * | 8 |
| Nguyen (2016) | * | * | * | * | - | * | * | * | * | 7 |
| Cheung (2024) | * | * | - | * | - | * | * | * | * | 7 |

Note: *: has been done/reported properly; -: has not been done/reported properly

# Supplementary Table 6. Risk of bias in population pharmacokinetic (PopPK) studies and physiologically based pharmacokinetic (PBPK) modelling

| First author, year | Participants | Predictor | Outcome | Analysis | Conclusion |
| --- | --- | --- | --- | --- | --- |
| Freiwald (2014) | + | + | + | + | Low Risk |
| Nakao (2019) | + | + | + | ? | Unclear Risk |
| Alsmadi (2021) | + | + | + | + | Low Risk |
| Gupta (2021a) | + | + | + | + | Low Risk |
| Hong (2017) | + | + | + | + | Low Risk |
| Wang (2016) | + | + | + | + | Low Risk |
| Chen (2021) | + | + | + | + | Low Risk |
| Oullet (2014) | + | + | + | + | Low Risk |
| Oullet (2016) | + | + | + | + | Low Risk |
| Xiong (2022) | + | + | + | + | Low Risk |
| Nguyen (2019) | + | + | + | + | Low Risk |
| Gerner (2021) | + | + | + | + | Low Risk |

Note: +: low risk of bias; ?: unclear risk of bias; -: high risk of bias

# Supplementary Table 7. Drug dosing in hepatic and renal impairment based on drug label and pharmacokinetic studies on impairment of renal and hepatic function.

| **Drug Name (dosage)** | **Hepatic Impairment Dosage** | **Renal Impairment Dosage** | **Hemodialysis / CAPD Dosage** | **Hepatotoxicity consideration** |
| --- | --- | --- | --- | --- |
| **Targeted therapy** | | | | |
| **EGFR Inhibitors** | | | | |
| Afatinib (40 mg orally once daily) | Package insert:   - Mild to moderate HI: no adjustment is needed. - Severe HI: has not been studied   PopPK Freiwald (2014):   - ALP 189 vs 106: 112 (108-117) % - ALP 509 vs 106: 122 (115-131) %   PopPK Nakao (2019):   - AST (25.0 vs 13.0): 81.9 vs 62.5 - AST (65.0 vs 13.0): 266.8 vs 62.5   Note: trough concentration at 8^th^ day  PK Schnell (2014):  PGM CP A vs control   - AUC_0–inf_: 92 (68–126) - C_max­_: 109 (82–144)   PGM CP B vs control   - AUC_0–inf_: 94 (72–124) - C_max­_: 126 (86–187) | Package insert:   - 15-29: 30 mg once daily - <15: has not been studied   PopPK Freiwald (2014):   - AUC PGM in eGFR 60 vs 79: 112 (109-115) - AUC PGM in eGFR 30 vs 79: 142 (130-154)   PopPK Nakao (2019):   - Renal impairment: NS   PK Weibe (2016):  PGM eGFR 30-59 vs >90   - AUC_0-inf_: 122 (96–155) - C_max­_: 101 (72–140)   PGM eGFR 15-29 vs >90:   - AUC_0-inf_: 150 (105–213) - C_max­_: 121 (90–163) | Package insert:  Dialysis: has not been studied  CR Yamaguchi (2015):  Trough concentration: 23.8 ng/mL  CS Imai (2016):  Trough concentration at 11^th^ day with initial dose of 30 mg: 37.2, 19.2, and 28.6 ng/mL | Package insert:  9,7% had liver test abnormalities, of which 0,2% were fatal   - Worsening liver function: withhold therapy - Severe hepatic impairment: discontinue treatment |
| Erlotinib (150 mg orally once daily) | Package insert:  Monitor patient with CP A, B, or C  Treatment should be stopped if total bilirubin > 3 x ULN  PK Miller (2007):  Direct bilirubin 1.0 to 7.0 mg/dL   - Half-life: 29.17 + 21.63 hours - Clearance: 2.37 + 1.10 L/h   AST > 3x ULN   - Half-life: 23.2 + 3.38 hours - Clearance: 1.88 + 0.19 L/h   PK O’Bryant (2012):  PGM CP B vs Control (erlotinib)   - AUC_0–t_: 92 (69-123) - C_max­_: 74 (56-95) - OSI-420 AUC and C_max_: NS   Case report Czejka (2013):   - OSI420 plasma concentration increased by 3x normal level in patient with total bilirubin 14.15 | Package insert:  No recommendation  PK Miller (2007):  Creatinine 2.5 to 5.0 mg/dL   - Half-life: 12.11 + 6.17 hours - Clearance: 5.30 + 3.24 L/h | Package insert: no recommendation  PK Togashi (2010):  HD erlotinib conc:   - AUC_0-24_: 23285 + 4358 - C_max_: 1614 + 177   Control erlotinib conc:   - AUC_0-24_: 44570 + 13923 - C_max_: 2434 + 689   HD OSI-420 conc:   - AUC_0-24_: 2094 + 670 - C_max_: 138 + 35   Control erlotinib conc:   - AUC_0-24_: 4827 + 3569 - C_max_: 241 + 163 | Package insert:   - Incidence of hepatic failure in the 3-monotherapy lung cancer was 0.4% - Incidence of hepatic failure in the pancreatic study was 0.4% - CP B: 10 out of 15 patients died within 30 days of the last dose - Patients without pre-existing hepatic impairment for total bilirubin levels greater than 3 times the upper limit of normal or transaminases greater than 5 times the upper limit of normal: withhold therapy - Patients with pre-existing hepatic impairment or biliary obstruction for doubling of bilirubin or tripling of transaminases values over baseline: withhold therapy - Patients with abnormal liver tests and do not improve significantly or resolve within three weeks: discontinue therapy |
| Dacomitinib (45 mg orally once daily) | Package insert:  No recommendation  PK Giri (2015):  PGM mild HI vs control   - AUC_0–inf_: 101 (73–138) - C_max­_: 103 (70–153)   PGM moderate HI vs Control   - AUC_0–inf_: 0.85 (62–115) - C_max­_: 80 (55–117)   PK Piscitelli (2022):  PGM severe HI vs control   - AUC_0–inf_: 104 (72–151) - C_max­_: 130 (86–199) | Package insert:  Normal dose in eGFR 30-89  No recommendation for eGFR <30 | Package insert:  No recommendation | Package insert: no information related to hepatotoxicity |
| Osimertinib (80 mg orally once daily) | Package insert:  mild to moderate HI: no adjustment is needed.  Severe HI: no recommendation  PK Grande (2019):  PGM CP A vs control   - AUC_0–inf_: 63 (47–85) - C_max­_: 51 (36–72)   PGM CP B vs control   - AUC_0–inf_: 68 (50–94) - C_max­_: 61 (42–89) | Package insert:  Normal dose in eGFR 15 - 89 mL/min  No recommendation for eGFR <15 mL/min  PK Vishwanathan (2020):  PGM eGFR <30 vs >90:   - AUC: 184 (93–363) - C_max­_: 119 (69–207)   PopPK Vishwanathan (2020):  PGM eGFR <30 vs >90:   - AUC_ss_: 126 (110–150)   PK Fujiwara (2023):  EGFR > 50, 80 mg   - AUC: 4310 ± 1870 - C_max­_: 619 ± 263   EGFR 30 – 50, 80 mg   - AUC: 3460 ± 2840 - C_max­_: 626 ± 353   EGFR <30 or HD, 40 mg   - AUC: 1880 ± 350 - C_max­_: 256 ± 42.5 | Package insert:  No recommendation  CR Matsunashi (2019):  Pre-HD  AUC_0=24_: 11797  C_max_: 672  Post-HD  AUC_0=24_: 11827  C_max_: 589  CR Tabata (2023):  C­_max_ 560 mg/week: 1,522 nM  C_max_ 480 mg/week: 592 nM | Package insert:  no information related to hepatotoxicity |
| Gefitinib (250 mg orally once daily) | Package insert:  Moderate to severe HI: monitor adverse reaction  PK single dose Horak (2011):  No impairment:   - AUC_0–t_: 1,415 (80.7) - C_max­_: 57.5 (99.5)   CP A cirrhosis   - AUC_0–t_: 1,980 (115.7) - C_max­_: 82.8 (109.7)   CP B cirrhosis   - AUC_0–t_: 5,137 (44.5) - C_max­_: 155.7 (52.9)   CP C cirrhosis   - AUC_0–t_: 5,137 (44.5) - C_max­_: 155.7 (52.9)   PK multiple dose Horak in liver metastasis (2011):  No impairment:   - AUC_SS(24)_: 8,896 (55) - C_ss­(max)_: 466.4 (52.6)   Moderate HI   - AUC_SS(24)_: 9,553 (66) - C_ss(max)­_: 517,7 (66.5)   Severe HI   - AUC_SS(24)_: 6,226 (24) - C_ss(max)­_: 371,8 (23.5) | Package insert:  RI: no clinical study has been conducted, consider to be safe | Package insert: no recommendation  CR Shinagawa (2007):  88.7% kept in plasma after HD  C_max_: 410.4  CR Luo (2016):  C_max_: 456 (non-HD days); 463 (HD days)  CR Yamaguchi (2015):  C­_max­_ on day 46: 609.2  C_CAPD fluid_: 34.6 | Package insert:   - 11.4% patients with increased ALT, 7.9% patients with increased AST, 2.7% patients with increased bilirubin - Incidence of fatal hepatotoxicity was 0.04% |
| **ALK Inhibitors** | | | | |
| Alectinib (600 mg orally twice daily) | Package insert:  mild to moderate HI: no adjustment is needed.  Severe HI: 450 mg orally twice daily  PK Grande (2019):  PGM alectinib in CP B vs control   - AUC_0–inf_: 160 (105-243) - C_max­_: 128 (86-188)   PGM alectinib in CP C vs control   - AUC_0–inf_: 220 (131-369) - C_max­_: 100 (55–183)   PGM M4 in CP B vs control   - AUC_0–inf_: 81 (50-130) - C_max­_: 65 (36-115)   PGM M4 in CP C vs control   - AUC_0–inf_: 66 (27-160) - C_max­_: 61 (27–139)   PBPK Alsmadi (2021):  AUC_0–inf_ control vs CP B vs CP C  3359 vs 3179 vs 4692  C_max_ control vs CP B vs CP C  127 vs 73 vs 78 | Package insert:  Normal dose in mild to moderate RI  No recommendation for severe RI | Package insert:  No recommendation  CR Park (2022)  C_max_ alectinib and M4 = 638 and 82 ng/mL  C_trough_ alectinib and M4: 562 and 66  Similar HD and non HD | Package insert:   - 4.6% patients with AST elevations greater than 5 times the upper limit of normal (ULN) in the first 3 months of treatment: temporarily withhold the therapy - 5.3% patients with ALT elevations greater than 5 times the ULN in the first 3 months of treatment: temporarily withhold the therapy - 3.7% patients with bilirubin elevations greater than 3 times the ULN in the first 3 months of treatment: temporarily withhold the therapy - Monitor liver function tests (ALT, AST, total bilirubin) every 2 weeks during the first 3 months of treatment |
| Brigatinib (90 mg orally once daily for 7 days, than increase to 180 mg) | Package insert:  mild to moderate HI: no adjustment is needed.  Severe HI: 60 mg for 7 days, then increase to 120 mg  PopPK Gupta (2021a); PGM AUC_ss_  Bilirubin 95% vs median: 97 (NS)  AST 95% vs median: 95 (NS)  ALT 95% vs median: 96 (NS)  PK Hanley (2023):  PGM CP A vs control   - AUC_0–inf_: 89 (70–114) - C_max­_: 94 (75–119)   PGM CP B vs control   - AUC_0–inf_: 99 (77–127) - C_max­_: 92 (73–116)   PGM CP C vs control   - AUC_0–inf_: 137 (107–176) - C_max­_: 165 (131–207) | Package insert:  eGFR 30-89: normal dose  eGFR 15-29: 60 mg for 7 days, then increase to 90 mg  eGFR <15: no recommendation  PopPK Gupta (2021a); AUC_ss_ PGM  Mild RI vs normal: 108 (NS)  Moderate RI vs normal: 106 (NS)  PK Gupta (2021b):  PGM eGFR <30 vs >90:   - AUC_0-inf_: 192 (152-242) - C_max­_: 114 (90–144) | Package insert:  No recommendation | Package insert:  AST elevations occurred in 38% of patients in the 90 mg group and 65% of patients in the 90→180 mg group. ALT elevations occurred in 34% of patients in the 90 mg group and 40% of patients in the 90→180 mg group. |
| Ceritinib (450 mg orally once daily) | Package insert:  mild HI: no adjustment is needed.  Moderate to severe HI: recommended dose has not been determined  PopPK Hong (2016); PGM AUC_ss_  Mild HI vs control: 103 (93-111) | Package insert:  No recommendation  PopPK Hong (2016); PGM AUC_ss_  Mild RI vs normal: 107 (99-114)  Moderate RI vs normal: 115 (101-130) | Package insert:  No recommendation | Package insert:   - 28% patients with ALT elevations > 5 x ULN - 16% patients with AST elevations > 5 x ULN   Monitor with liver laboratory tests (ALT, AST, total bilirubin) |
| Crizotinib (250 mg orally twice daily) | Package insert:  Mild HI: no adjustment is needed  Moderate HI: 200 mg twice daily  Severe HI: 250 mg once daily  PopPK Wang (2016); PGM AUC_ss_  Bilirubin 2.1 vs 0.41: 109 (102-121)  AST (7-124): NS  PK El-Khoueiry (2018):  PGM mild HI (250 mg BID) vs control   - AUC_0–inf_: 91 (57–147) - C_max­_: 94 (57–145)   PGM moderate HI (200 mg BID) vs control   - AUC_daily_: 114 (73–177) - C_max­_: 109 (70–169)   PGM severe HI (250 mg QD) vs control   - AUC_daily_: 65 (40–106) - C_max­_: 73 (49–108) | Package insert:  eGFR 30-89: normal dose  eGFR <30: 250 mg once daily  PopPK Wang (2016); PGM AUC_ss_  Mild RI vs normal: 106 (101-111)  Moderate RI vs normal: 118 (113-124)  PK Tan (2017):  PGM eGFR <30 vs >90:   - AUC_0-inf_: 180 (129-254) - C_max­_: 134 (99–182)   PBPK Tan (2017):  PGM 250 mg QD multiple dose   - AUC_0-inf_: 154 - C_max­_: 153 | Package insert:  No recommendation | Package insert:   - 0.1% patients with fatal hepatotoxicity - 11% patients with increased ALT > 5 x ULN - 6% patients with increased AST > 5 x ULN   Monitor liver function tests (ALT, AST, total bilirubin) every 2 weeks during the first 2 months, then once a month, and as clinically indicated   - ALT/AST > 5 x ULN with total bilirubin less than or equal to 1.5 x ULN: withhold the therapy - ALT/AST > 3 x ULN with concurrent total bilirubin > 1.5 x ULN in the absence of cholestasis or hemolysis: permanently discontinue |
| Lorlatinib (100 mg orally one daily) | Package insert:  Mild HI: no adjustment is needed  Moderate to severe HI: no recommendation  PopPK Chen (2021) steady-state clearance; mean (SD) in L/h  Normal: 13.78 (2.55)  Mild HI: 13.71 (2.98) | Package insert:  eGFR 30-89: normal dose  eGFR <30: no recommendation  PopPK Chen (2021) steady-state clearance; mean (SD) in L/h  Normal: 15.21 (2.52)  eGFR 60-90:12.90 (1.80)  eGFR 30-60: 11.50 (1.66)  eGFR <30: 7.68  PK Lin (2022)  PGM eGFR 60-90 vs >90:   - AUC_0-inf_: 104 (80-136)   PGM eGFR 30-60 vs >90:   - AUC_0-inf_: 119 (91-154)   PGM eGFR 30-60 vs >90:   - AUC_0-inf_: 141 (98-204) | Package insert:  No recommendation | Package insert:  In 10 of 12 subjects with severe hepatotoxicity  Grade 4 ALT/AST elevations in 50% subjects  Grade 3 ALT/AST elevations in 33% subjects  Grade 2 ALT/AST elevations in 8% subjects |
| **BRAF/MEK Inhibitors** | | | | |
| Dabrafenib (150 mg orally twice daily) | Package insert:  Mild HI: no adjustment is needed  Moderate to severe HI: increased dabrafenib exposure, an appropriate dose has not been established  PopPK Ouellet (2014): PGM Cl/F  Mild and moderate HI: 102 (93-112) | Package insert:  No recommendation  PopPK Ouellet (2014): PGM Cl/F  eGFR 60-90: 95 (90-100)  eGFR 30-60: 92 (83-102) | Package insert:  No recommendation  CR Park (2017):  Dabrafenib 75 mg once daily  HD did not lower the plasma concentration of dabrafenib | Package insert:  no information related to hepatotoxicity |
| Trametinib (2 mg orally once daily) | Package insert:  Mild HI: no adjustment is needed  Moderate to severe HI: no recommendation  PopPK Ouellet (2016): PGM Cl/F  Mild and moderate HI: 102 (92-112)  PK Voon (2022); dose normalized to 2 mg  Normal group GM  C_max_: 26.2 (31.4) AUC_0-24_: 449.5 (28.1)  Mild HI GM  C_max_: 26.2 (53.8) AUC_0-24_: 352.1 (51.9)  Moderate HI GM  C_max_: 16.8 (12.6) AUC_0-24_: 311.0 (8.1)  Severe HI GM  C_max_: 15.3 (43.6) AUC_0-24_: 228.9 (36.4) | Package insert:  No recommendation  PopPK Ouellet (2016): PGM Cl/F  eGFR 60-90: 94 (87-101)  eGFR 30-60: 96 (78-113) | Package insert:  No recommendation  CR Park (2017):  Conc during dialysis = 2.4–3.5 ng/ml | Package insert:  no information related to hepatotoxicity |
| Vemurafenib (960 mg orally twice daily) | Package insert:  Mild to moderate HI: no adjustment is needed  Severe HI: no recommendation | Package insert:  eGFR 30-89: normal dose  eGFR <30: no recommendation | Package insert:  No recommendation | Package insert:  Monitor ALT, AST, and total bilirubin before treatment and monthly during treatment, or as clinically indicated   - Grade 4 adverse reaction: permanently discontinue - Grade 2 or greater adverse reaction: withhold the therapy   Grade 0-1 adverse reaction: 720 mg twice daily in patients with grade 2 or 3 for first appearance, 480 mg twice daily in patients with grade 2 or 3 for second appearance or grade 4 for first appearance |
| **NTRK Inhibitors** | | | | |
| Larotrectinib (100 mg orally twice daily) | Package insert:  Mild HI: no adjustment is needed  Moderate to HI: reduce to 50% | Package insert: normal dose | Package insert:  No recommendation | Package insert:   - 52% patients with increased AST, of which 3.1% were grade 3-4 - 45% patients with increased ALT, of which 2.5% were grade 3-4   Monitor liver function tests (ALT, AST, ALP, and bilirubin) before initiation and monitor every 2 weeks during the first 2 months, then monthly, or as clinically indicated   - AST/ALT ≥ 5 x ULN with total bilirubin ≤ 2 x ULN: withhold the therapy   AST/ALT > 3 x ULN with total bilirubin > 2 x ULN in the absence of alternative causes: permanently discontinue |
| Entrectinib (600 mg orally once daily) | Package insert:  Mild HI: no adjustment is needed  Moderate to severe HI: no recommendation | Package insert:  eGFR 30-89: normal dose  eGFR <30: no recommendation | Package insert:  No recommendation | Package insert:   - 42% patients with increased AST, of which 2.5% were grade 3-4 - 36% patients with increased ALT, of which 2.8% were grade 3-4   Monitor liver function tests every 2 weeks during the first month, then monthly, or as clinically indicated   - The reduction of the dose based on the starting dose once daily   Patients unable to tolerate the therapy after two dose reductions: permanently discontinue |
| **MET Inhibitors** | | | |  |
| Capmatinib (400 mg orally twice daily) | Package insert: no recommendation  PK Chen (2022):  PGM CP A vs control   - AUC_0–inf_: 77 (53–111) - C_max­_: 72 (48–110)   PGM CP B vs control   - AUC_0–inf_: 91 (62–128) - C_max­_: 83 (56–122)   PGM CP C vs control   - AUC_0–inf_: 124 (86–178) - C_max­_: 102 (86–178) | Package insert:  eGFR 30-89: normal dose  eGFR <30: no recommendation | Package insert:  No recommendation | Package insert:   - 15% patients with increased ALT/AST, of which 7% were grade 3 or 4   Monitor liver function tests before treatment, every 2 weeks during the first 3 months of treatment, then monthly or as clinically indicated   - Increased ALT/AST without increased total bilirubin grade 3 (withhold the therapy) or grade 4 (permanently discontinue) - Increased ALT/AST with increased total bilirubin in the absence of cholestasis or hemolysis: permanently discontinue - Increased total bilirubin without concurrent increased ALT/AST grade 2-3 (withhold the therapy) or grade 4 (permanently discontinue) |
| Tepotinib (450 mg orally once daily) | Package insert:  Mild to moderate HI: no adjustment is needed  Severe HI: no recommendation  PopPK Xiong (2022):   - moderate HI had 13% lower total plasma AUC | Package insert:  eGFR 30-89: normal dose  eGFR <30: no recommendation  PopPK Xiong (2022); PGM AUC_ss_   - 39.4 – 59 vs 99.8: 102 (93-112) | Package insert:  No recommendation | Package insert:   - 18% patients with increased ALT/AST, of which 4.7% were grade 3-4   Monitor liver function tests before treatment, every 2 weeks during the first 3 months of treatment, then monthly or as clinically indicated   - Increased ALT/AST without increased total bilirubin grade 3 (withhold the therapy) or grade 4 (permanently discontinue) - Increased ALT/AST with increased total bilirubin in the absence of cholestasis or hemolysis: permanently discontinue - Increased total bilirubin without concurrent increased ALT/AST grade 3 (withhold the therapy) or grade 4 (permanently discontinue) |
| **RET Inhibitors** | | | | |
| Cabozantinib (60 mg orally once daily) | Package insert:   - Mild HI: normal dose - Moderate HI: reduce the dose from 60 mg daily to 40 mg daily - Severe HI has not been studied   PK Nguyen (2016):  PGM CP A vs control   - AUC_0–inf_: 181 (121–270) - C_max­_: 110 (82–148)   PGM CP B vs control   - AUC_0–inf_: 163 (107–246) - C_max­_: 70 (53–95)   PopPK Nguyen (2019): PGM Cl/F   - Mild HI: 112 (106-118) - Moderate HI: 98 (78-122)   PBPK Gerner (2021):  Mild HI:   - AUC­_0-t­_ increased by 64% - Mean predicted C_max­_ slightly increased   Moderate HI:   - AUC­_0-t­_ increased by 50% - Mean predicted C_max_ slightly lowered | Package insert:   - eGFR 30-89: normal dose - eGFR<30: has not been studied   PK Nguyen (2016):  PGM eGFR 60-90 vs >90:   - AUC_0-inf_: 130 (99-171) - C_max­_: 119 (92–156)   PGM eGFR 30-60 vs >90:   - AUC_0-inf_: 106 (80-140) - C_max­_: 103 (79–134) | Package insert:  No recommendation  CR Zimmermann (2021):  AUC_trough­_ in HD: 348 (278-430)  AUC_trough­_ in control: 1375 (601-2602) | Package insert:   - Monitor liver enzymes before treatment and periodically throughout treatment - 11% patients with increased ALT/AST grade 3 and 4 - Withhold the therapy for: intolerable grade 2 adverse reactions, grade 3 or 4 adverse reactions, osteonecrosis of the jaw. Then, upon resolution, reduce the dose |
| Selpercatinib (less than 50 kg: 120 mg orally twice daily; 50 kg or greater: 160 mg orally twice daily) | Package insert:   - Mild or moderate HI: no dose adjustment - Severe hepatic impairment: 80 mg orally twice daily | Package insert:   - eGFR 15-89: no dose adjustment | Package insert:  No recommendation | Package insert:   - 59% patients with increased AST, of which 11% were grade 3 or 4: withhold the therapy - 55% patients with increased ALT, of which 12% were grade 3 or 4: withhold the therapy - Monitor ALT and AST before treatment, every 2 weeks during the first 3 months, then monthly, or as clinically indicated |
| Pralsetinib (400 mg orally once daily) | Package insert:   - Mild HI: no dose adjustment - Moderate to severe HI: has not been studied   PK Cheung (2024):  PGM CP B vs control   - AUC_0–inf_: 112 (65–193) - C_max­_: 99 (60–163)   PGM CP C vs control   - AUC_0–inf_: 86 (51–144) - C_max­_: 69 (35–131) | Package insert:  No recommendation | Package insert:  No recommendation | Package insert:   - 49% patients with increased AST, of which 7% were grade 3 or 4: withhold the therapy - 37% patients with increased ALT, of which 4.8% were grade 3 or 4: withhold the therapy - Monitor AST and ALT before treatment, every 2 weeks during the first 3 months, then monthly or as clinically indicated |
| **KRAS Inhibitor** | | | | |
| Sotorasib (960 mg orally once daily) | Package insert: No recommendation | Package insert: No recommendation | Package insert:  No recommendation | Package insert:   - 18% patients with ALT/AST elevations, of which 6% were grade 3 and 0.6% were grade 4   Monitor liver function tests (ALT, AST, and total bilirubin) before treatment, every 3 weeks for the first 3 months, then monthly or as clinically indicated   - Grade 2 AST/ALT with symptoms or grade 3 or 4 AST/ALT: withhold the therapy - AST/ALT > 3 x ULN with total bilirubin > 2 x ULN in the absence of alternative causes: permanently discontinue |

Note: HI: hepatic impairment; PopPK: population pharmacokinetic study; ALP: alkaline phosphatase; ALT: alanine transaminase; AST: alanine aminotransferase; PK: pharmacokinetic study; GM: geometric mean; PGM: percent geometric mean; AUC: area under the concentration on-time curve; AUC_0-inf_ = AUC from time 0 extrapolated to infinity; AUC_ss_ = AUC on steady state; C_max_: maximum serum concentration; CR: case report; CP: child pugh; NS: nonsignificant; ULN: upper limit normal; HD: hemodialysis; CAPD: continuous ambulatory peritoneal dialysis; -PBPK: physiologically based pharmacokinetic modelling; AUC were noted by ng h/mL; C_max_ were noted by ng/mL; PGM stated in % (90% CI). Package insert data were searched on May 2^nd^, 2024.

# Supplementary Table 8. Dose adjustment for oral targeted therapy in hepatic impairment population

| **Certainty assessment** | | | | | | | **Summary of findings** |
| --- | --- | --- | --- | --- | --- | --- | --- |
| **Participants (studies) Follow-up** | **Risk of bias** | **Inconsistency** | **Indirectness** | **Imprecision** | **Publication bias** | **Overall certainty of evidence** |  |
| **Gefitinib in hepatic impairment population** | | | | | | | |
| 81 (2 non-randomised studies) | not serious | serious^a^ | not serious | not serious | none | ⨁◯◯◯ Very low^a^ | For cirrhosis with moderate to severe hepatic impairment, close monitor should be considered. |
| **Trametinib in hepatic impairment population** | | | | | | | |
| 46 (1 non-randomised study) | not serious | not serious | serious^b^ | serious^c^ | none | ⨁◯◯◯ Very low^b,c^ | Trametinib can be administered at 1.5 mg QD as starting dose in moderate hepatic impairment.  Trametinib can be administered at 1 mg QD as starting dose in severe hepatic impairment |
| **Capmatinib in hepatic impairment population** | | | | | | | |
| 29 (1 non-randomised study) | not serious | not serious | not serious | not serious | none | ⨁⨁◯◯ Low | Capmatinib can be administered at normal dose (400 mg BID) in any degree of hepatic impairment |
| **Pralsetinib in hepatic impairment population** | | | | | | | |
| 29 (1 non-randomised study) | not serious | not serious | not serious | not serious | none | ⨁⨁◯◯ Low | Pralsetinib can be administered at normal dose (400 mg QD) in any degree of hepatic impairment |

Explanations

a. Cirrhosis subjects were given only a single dose, whereas patients with liver metastasis were given multiple dose

b. Using various solid tumor

c. Low number of subject with moderate and severe hepatic impairment

# Supplementary Table 9. Dose adjustment for oral targeted therapy in renal impairment population

| **Certainty assessment** | | | | | | | **Summary of findings** |
| --- | --- | --- | --- | --- | --- | --- | --- |
| **Participants (studies) Follow-up** | **Risk of bias** | **Inconsistency** | **Indirectness** | **Imprecision** | **Publication bias** | **Overall certainty of evidence** |  |
| **Erlotinib in renal impairment population** | | | | | | | |
| 55 (1 non-randomised study) | not serious | not serious | not serious | not serious | dose response gradient | ⨁⨁⨁◯ Moderate | Erlotinib can be administered at normal dose (150 mg QD) in any degree of renal impairment |
| **Osimertinib in renal impairment population** | | | | | | | |
| 48 (2 non-randomised studies) | not serious | not serious | not serious | not serious | dose response gradient | ⨁⨁⨁◯ Moderate | Osimertinib can be administered at normal dose (80 mg QD) in any degree of renal impairment |
| **Alectinib in renal impairment population** | | | | | | | |
| 0 cases 0 controls (1 non-randomised study) | not serious | not serious | not serious | serious^a^ | none | ⨁◯◯◯ Very low^a^ | Alectinib can be administered at normal dose (600 mg BID) in any degree of renal impairment |
| **Brigatinib in renal impairment population** | | | | | | | |
| 16 (1 non-randomised study) | not serious | not serious | not serious | not serious | none | ⨁⨁◯◯ Low | Brigatinib can be administered at 60 mg QD as starting dose in patients with eGFR 10-29 mL/min/1.73m2 |
| **Ceritinib in renal impairment population** | | | | | | | |
| 0 cases 0 controls (1 non-randomised study) | not serious | not serious | not serious | serious^b^ | none | ⨁◯◯◯ Very low^b^ | Ceritinib can be administered at normal dose (450 mg QD) in patients with eGFR >30 mL/min/1.73m2 |
| **Lorlatanib in renal impairment population** | | | | | | | |
| 29 (1 non-randomised study) | not serious | not serious | not serious | not serious | none | ⨁⨁◯◯ Low | Lorlatanib can be administered at 75 mg QD in patients with eGFR <30 mL/min/1.73m2 |
| **Dabrafenib in renal impairment population** | | | | | | | |
| 0 cases 0 controls (1 non-randomised study) | not serious | not serious | serious^c^ | serious^b^ | none | ⨁◯◯◯ Very low^b,c^ | Dabrafenib can be administered at normal dose (150 mg BID) in patients with eGFR >30 mL/min/1.73m2 |
| **Trametinib in renal impairment population** | | | | | | | |
| 0 cases 0 controls (1 non-randomised study) | not serious | not serious | serious^c^ | serious^b^ | none | ⨁◯◯◯ Very low^b,c^ | Trametinib can be administered at normal dose (2 mg QD) in patients with eGFR >30 mL/min/1.73m2 |
| **Tepotinib in renal impairment population** | | | | | | | |
| 0 cases 0 controls (1 non-randomised study) | not serious | not serious | not serious | serious^b^ | dose response gradient | ⨁⨁◯◯ Low^b^ | Tepotinib can be administered at normal dose (450 mg QD) in patients with eGFR >40 mL/min/1.73m2 |

Explanations

a. Using PBPK study

b. Using PopPK study

c. Using melanoma population

# Supplementary Table 10. Dose adjustment for oral targeted therapy in HD/CAPD population

| **Certainty assessment** | | | | | | | **Summary of findings** |
| --- | --- | --- | --- | --- | --- | --- | --- |
| **Participants (studies) Follow-up** | **Risk of bias** | **Inconsistency** | **Indirectness** | **Imprecision** | **Publication bias** | **Overall certainty of evidence** |  |
| **Afatinib in HD population** | | | | | | | |
| 4 (2 non-randomised studies) | not serious | not serious | not serious | not serious | none | ⨁⨁◯◯ Low | Afatinib can be administered at 30 mg QD in HD population |
| **Erlotinib in HD population** | | | | | | | |
| 8 (1 non-randomised study) | not serious | not serious | not serious | not serious | none | ⨁⨁◯◯ Low | Erlotinib can be administered at the standard dosage of 150 mg QD in the HD population. |
| **Osimertinib in HD population** | | | | | | | |
| 33 (2 non-randomised studies) | not serious | not serious | not serious | not serious | dose response gradient | ⨁⨁⨁◯ Moderate | Osimertinib can be administered at the standard dosage of 80 mg QD as the starting dose in the HD population. |
| **Gefitinib in HD and CAPD population** | | | | | | | |
| 3 (3 non-randomised studies) | not serious | not serious | not serious | not serious | none | ⨁⨁◯◯ Low | Gefitinib can be administered at the standard dosage of 250 mg QD in the HD/CAPD population. |
| **Dabrafenib and Trametinib in HD population** | | | | | | | |
| 1 (1 non-randomised study) | not serious | not serious | serious^a^ | not serious | none | ⨁◯◯◯ Very low^a^ | Dabrafenib and Trametinib combination should be started as low as possible (75 mg/0.5 mg QD) |
| **Alectinib in HD population** | | | | | | | |
| 1 (1 non-randomised study) | not serious | not serious | not serious | not serious | none | ⨁⨁◯◯ Low | Alectinib can be administered at the standard dose of 600 mg BID in the HD population |

Explanations

a. Using a melanoma patients

References

1. Wiebe S, Schnell D, Külzer R, Gansser D, Weber A, Wallenstein G, et al. Influence of Renal Impairment on the Pharmacokinetics of Afatinib: An Open-Label, Single-Dose Study. Eur J Drug Metab Pharmacokinet. 2017;42: 461–469. doi:10.1007/s13318-016-0359-9

2. Schnell D, Buschke S, Fuchs H, Gansser D, Goeldner R-G, Uttenreuther-Fischer M, et al. Pharmacokinetics of afatinib in subjects with mild or moderate hepatic impairment. Cancer Chemother Pharmacol. 2014;74: 267–275. doi:10.1007/s00280-014-2484-y

3. Freiwald M, Schmid U, Fleury A, Wind S, Stopfer P, Staab A. Population pharmacokinetics of afatinib, an irreversible ErbB family blocker, in patients with various solid tumors. Cancer Chemother Pharmacol. 2014;73: 759–770. doi:10.1007/s00280-014-2403-2

4. Nakao K, Kobuchi S, Marutani S, Iwazaki A, Tamiya A, Isa S, et al. Population pharmacokinetics of afatinib and exposure-safety relationships in Japanese patients with EGFR mutation-positive non-small cell lung cancer. Sci Rep. 2019;9: 18202. doi:10.1038/s41598-019-54804-9

5. Yamaguchi T, Hayashi H, Isogai S, Hayashi M, Uozu S, Goto Y, et al. Afatinib administration in a patient with non-small cell lung cancer harboring uncommon EGFR mutation G719A undergoing hemodialysis. Cancer Treat Commun. 2015;4: 169–171. doi:10.1016/j.ctrc.2015.09.006

6. Imai H, Kaira K, Naruse I, Hayashi H, Iihara H, Kita Y, et al. Successful afatinib treatment of advanced non-small-cell lung cancer patients undergoing hemodialysis. Cancer Chemother Pharmacol. 2017;79: 209–213. doi:10.1007/s00280-016-3201-9

7. Miller AA, Murry DJ, Owzar K, Hollis DR, Lewis LD, Kindler HL, et al. Phase I and Pharmacokinetic Study of Erlotinib for Solid Tumors in Patients With Hepatic or Renal Dysfunction: CALGB 60101. Journal of Clinical Oncology. 2007;25: 3055–3060. doi:10.1200/JCO.2007.11.6210

8. O’Bryant CL, Haluska P, Rosen L, Ramanathan RK, Venugopal B, Leong S, et al. An open-label study to describe pharmacokinetic parameters of erlotinib in patients with advanced solid tumors with adequate and moderately impaired hepatic function. Cancer Chemother Pharmacol. 2012;69: 605–612. doi:10.1007/s00280-011-1733-6

9. Czejka M, Sahmanovic A, Buchner P, Steininger T, Dittrich C. Disposition of Erlotinib and Its Metabolite OSI420 in a Patient with High Bilirubin Levels. Case Rep Oncol. 2013;6: 602–608. doi:10.1159/000357211

10. Togashi Y, Masago K, Fukudo M, Terada T, Ikemi Y, Kim YH, et al. Pharmacokinetics of Erlotinib and Its Active Metabolite OSI-420 in Patients with Non-small Cell Lung Cancer and Chronic Renal Failure Who Are Undergoing Hemodialysis. Journal of Thoracic Oncology. 2010;5: 601–605. doi:10.1097/JTO.0b013e3181d32287

11. Horak J, White J, Harris AL, Verrill M, Carmichael J, Holt A, et al. The effect of different etiologies of hepatic impairment on the pharmacokinetics of gefitinib. Cancer Chemother Pharmacol. 2011;68: 1485–1495. doi:10.1007/s00280-011-1611-2

12. Shinagawa N, Yamazaki K, Asahina H, Agata J, Itoh T, Nishimura M. Gefitinib administration in a patient with lung cancer undergoing hemodialysis. Lung Cancer. 2007;58: 422–424. doi:10.1016/j.lungcan.2007.06.001

13. Luo J, Ni L, Wang M, Zhong W, Xiao Y, Zheng K, et al. Pharmacokinetic analysis of gefitinib in a patient with advanced non‐small cell lung cancer undergoing hemodialysis. Thorac Cancer. 2016;7: 251–253. doi:10.1111/1759-7714.12263

14. Yamaguchi T, Isogai S, Okamura T, Uozu S, Mieno Y, Hoshino T, et al. Pharmacokinetics of Gefitinib in a Patient with Non-Small Cell Lung Cancer Undergoing Continuous Ambulatory Peritoneal Dialysis. Case Rep Oncol. 2015;8: 78–82. doi:10.1159/000375485

15. Giri N, Masters JC, Plotka A, Liang Y, Boutros T, Pardo P, et al. Investigation of the impact of hepatic impairment on the pharmacokinetics of dacomitinib. Invest New Drugs. 2015;33: 931–941. doi:10.1007/s10637-015-0256-0

16. Piscitelli J, Chen J, LaBadie RR, Salageanu J, Chung C-H, Tan W. The Effect of Hepatic Impairment on the Pharmacokinetics of Dacomitinib. Clin Drug Investig. 2022;42: 221–235. doi:10.1007/s40261-022-01125-x

17. Grande E, Harvey RD, You B, Batlle JF, Galbraith H, Sarantopoulos J, et al. Pharmacokinetic Study of Osimertinib in Cancer Patients with Mild or Moderate Hepatic Impairment. Journal of Pharmacology and Experimental Therapeutics. 2019;369: 291–299. doi:10.1124/jpet.118.255919

18. Vishwanathan K, Sanchez‐Simon I, Keam B, Penel N, de Miguel‐Luken M, Weilert D, et al. A multicenter, phase I, pharmacokinetic study of osimertinib in cancer patients with normal renal function or severe renal impairment. Pharmacol Res Perspect. 2020;8. doi:10.1002/prp2.613

19. Fujiwara Y, Makihara R, Hase T, Hashimoto N, Naito T, Tsubata Y, et al. Pharmacokinetic and dose‐finding study of osimertinib in patients with impaired renal function and low body weight. Cancer Sci. 2023;114: 2087–2097. doi:10.1111/cas.15736

20. Matsunashi A, Fujimoto D, Hosoya K, Irie K, Fukushima S, Tomii K. Osimertinib in a patient with non-small cell lung cancer and renal failure undergoing hemodialysis: a case report. Invest New Drugs. 2020;38: 1192–1195. doi:10.1007/s10637-019-00851-y

21. Tabata K, Aoki M, Miyata R, Umehara T, Harada-Takeda A, Kamimura G, et al. Successful Treatment with Osimertinib Based on Therapeutic Drug Monitoring in a Hemodialysis Patient with Non-Small Cell Lung Cancer: A Case Report. Case Rep Oncol. 2023; 705–710. doi:10.1159/000531840

22. Morcos PN, Cleary Y, Sturm‐Pellanda C, Guerini E, Abt M, Donzelli M, et al. Effect of Hepatic Impairment on the Pharmacokinetics of Alectinib. The Journal of Clinical Pharmacology. 2018;58: 1618–1628. doi:10.1002/jcph.1286

23. Alsmadi MM, AL‐Daoud NM, Jaradat MM, Alzughoul SB, Abu Kwiak AD, Abu Laila SS, et al. Physiologically‐based pharmacokinetic model for alectinib, ruxolitinib, and panobinostat in the presence of cancer, renal impairment, and hepatic impairment. Biopharm Drug Dispos. 2021;42: 263–284. doi:10.1002/bdd.2282

24. Park JE, Yoon Y, Kim CH, Lee J. Pharmacokinetics of alectinib and its metabolite <scp>M4</scp> in a patient with advanced lung adenocarcinoma undergoing hemodialysis: A case report. Thorac Cancer. 2022;13: 1224–1226. doi:10.1111/1759-7714.14357

25. Gupta N, Wang X, Offman E, Prohn M, Narasimhan N, Kerstein D, et al. Population Pharmacokinetics of Brigatinib in Healthy Volunteers and Patients With Cancer. Clin Pharmacokinet. 2021;60: 235–247. doi:10.1007/s40262-020-00929-4

26. Gupta N, Hanley MJ, Kerstein D, Tugnait M, Narasimhan N, Marbury TC, et al. Effect of severe renal impairment on the pharmacokinetics of brigatinib. Invest New Drugs. 2021;39: 1306–1314. doi:10.1007/s10637-021-01095-5

27. Hanley MJ, Kerstein D, Tugnait M, Narasimhan N, Marbury TC, Venkatakrishnan K, et al. Brigatinib pharmacokinetics in patients with chronic hepatic impairment. Invest New Drugs. 2023;41: 402–410. doi:10.1007/s10637-023-01339-6

28. Hong Y, Passos VQ, Huang P, Lau YY. Population Pharmacokinetics of Ceritinib in Adult Patients With Tumors Characterized by Genetic Abnormalities in Anaplastic Lymphoma Kinase. The Journal of Clinical Pharmacology. 2017;57: 652–662. doi:10.1002/jcph.849

29. Wang E, Nickens DJ, Bello A, Khosravan R, Amantea M, Wilner KD, et al. Clinical Implications of the Pharmacokinetics of Crizotinib in Populations of Patients with Non–Small Cell Lung Cancer. Clinical Cancer Research. 2016;22: 5722–5728. doi:10.1158/1078-0432.CCR-16-0536

30. El-Khoueiry AB, Sarantopoulos J, O’Bryant CL, Ciombor KK, Xu H, O’Gorman M, et al. Evaluation of hepatic impairment on pharmacokinetics and safety of crizotinib in patients with advanced cancer. Cancer Chemother Pharmacol. 2018;81: 659–670. doi:10.1007/s00280-018-3517-8

31. Tan W, Yamazaki S, Johnson TR, Wang R, O’Gorman MT, Kirkovsky L, et al. Effects of Renal Function on Crizotinib Pharmacokinetics: Dose Recommendations for Patients with ALK-Positive Non-Small Cell Lung Cancer. Clin Drug Investig. 2017;37: 363–373. doi:10.1007/s40261-016-0490-z

32. Chen J, O’Gorman MT, James LP, Klamerus KJ, Mugundu G, Pithavala YK. Pharmacokinetics of Lorlatinib After Single and Multiple Dosing in Patients with Anaplastic Lymphoma Kinase (ALK)-Positive Non-Small Cell Lung Cancer: Results from a Global Phase I/II Study. Clin Pharmacokinet. 2021;60: 1313–1324. doi:10.1007/s40262-021-01015-z

33. Lin S, Gong J, Canas GC, Winkle P, Pelletier K, LaBadie RR, et al. A Phase I Study to Evaluate the Pharmacokinetics and Safety of Lorlatinib in Adults with Mild, Moderate, and Severe Renal Impairment. Eur J Drug Metab Pharmacokinet. 2022;47: 235–245. doi:10.1007/s13318-021-00747-4

34. Ouellet D, Gibiansky E, Leonowens C, O’Hagan A, Haney P, Switzky J, et al. Population pharmacokinetics of dabrafenib, a BRAF inhibitor: Effect of dose, time, covariates, and relationship with its metabolites. The Journal of Clinical Pharmacology. 2014;54: 696–706. doi:10.1002/jcph.263

35. Park JJ, Boddy A V., Liu X, Harris D, Lee V, Kefford RF, et al. Pharmacokinetics of dabrafenib in a patient with metastatic melanoma undergoing haemodialysis. Pigment Cell Melanoma Res. 2017;30: 68–71. doi:10.1111/pcmr.12557

36. Ouellet D, Kassir N, Chiu J, Mouksassi M-S, Leonowens C, Cox D, et al. Population pharmacokinetics and exposure–response of trametinib, a MEK inhibitor, in patients with BRAF V600 mutation-positive melanoma. Cancer Chemother Pharmacol. 2016;77: 807–817. doi:10.1007/s00280-016-2993-y

37. Voon PJ, Chen EX, Chen HX, Lockhart AC, Sahebjam S, Kelly K, et al. Phase I pharmacokinetic study of single agent trametinib in patients with advanced cancer and hepatic dysfunction. Journal of Experimental & Clinical Cancer Research. 2022;41: 51. doi:10.1186/s13046-021-02236-7

38. Chen X, Cui X, Pognan N, Quinlan M, Kapoor S, Rahmanzadeh G, et al. Pharmacokinetics of capmatinib in participants with hepatic impairment: A phase 1, open‐label, single‐dose, parallel‐group study. Br J Clin Pharmacol. 2022;88: 91–102. doi:10.1111/bcp.14929

39. Xiong W, Papasouliotis O, Jonsson EN, Strotmann R, Girard P. Population pharmacokinetic analysis of tepotinib, an oral MET kinase inhibitor, including data from the VISION study. Cancer Chemother Pharmacol. 2022;89: 655–669. doi:10.1007/s00280-022-04423-5

40. Nguyen L, Chapel S, Tran BD, Lacy S. Updated Population Pharmacokinetic Model of Cabozantinib Integrating Various Cancer Types Including Hepatocellular Carcinoma. The Journal of Clinical Pharmacology. 2019;59: 1551–1561. doi:10.1002/jcph.1467

41. Nguyen L, Holland J, Ramies D, Mamelok R, Benrimoh N, Ciric S, et al. Effect of Renal and Hepatic Impairment on the Pharmacokinetics of Cabozantinib. The Journal of Clinical Pharmacology. 2016;56: 1130–1140. doi:10.1002/jcph.714

42. Gerner B, Scherf-Clavel O. Physiologically Based Pharmacokinetic Modelling of Cabozantinib to Simulate Enterohepatic Recirculation, Drug–Drug Interaction with Rifampin and Liver Impairment. Pharmaceutics. 2021;13: 778. doi:10.3390/pharmaceutics13060778

43. Zimmermann S, Kurlbaum M, Mayer S, Fassnacht M, Kroiss M, Scherf-Clavel O. Simulation-Based Interpretation of Therapeutically Monitored Cabozantinib Plasma Concentration in Advanced Adrenocortical Carcinoma with Hemodialysis. Ther Drug Monit. 2021;43: 706–711. doi:10.1097/FTD.0000000000000905

44. Cheung KWK, Tang Y, Anders D, Barata T, Scalori A, Agarwal P, et al. Exploring the Impact of Hepatic Impairment on Pralsetinib Pharmacokinetics. Pharmaceutics. 2024;16: 564. doi:10.3390/pharmaceutics16040564
