# Supplementary material for: Tailoring oral targeted therapies dosage in lung cancer: A systematic review of pharmacokinetics studies on renal and hepatic impairment
Source: PLoS One. 2025 Jul 29;20(7):e0324056. doi: 10.1371/journal.pone.0324056 (PMC12306784; doi:10.1371/journal.pone.0324056)
Supplement: Attachment — Submitted filename: Comments.pdf [file pone.0324056.s004.pdf]

There are minor inconsistencies in your statistical analysis. Great work overall, but kindly ensure to include figures that explains your statistical analysis.
