# Supplementary material for: Tailoring oral targeted therapies dosage in lung cancer: A systematic review of pharmacokinetics studies on renal and hepatic impairment
Source: PLoS One. 2025 Jul 29;20(7):e0324056. doi: 10.1371/journal.pone.0324056 (PMC12306784; doi:10.1371/journal.pone.0324056)
Supplement: Attachment — Submitted filename: Review_2.pdf [file pone.0324056.s006.pdf]

The review is thorough, covering a broad range of oral targeted therapies for lung cancer in patients with hepatic and renal impairment.

Table 1 which talks about summary of dose adjustments is critical but lacks an interpretation section**.**

Figures 3 could be referenced more explicitly in the discussion. (Instead of saying "**Key findings are summarized in Fig. 3**," specifying which insights the figure conveys makes more sense in my opinion.)
