# Supplementary material for: Tailoring oral targeted therapies dosage in lung cancer: A systematic review of pharmacokinetics studies on renal and hepatic impairment
Source: PLoS One. 2025 Jul 29;20(7):e0324056. doi: 10.1371/journal.pone.0324056 (PMC12306784; doi:10.1371/journal.pone.0324056)
Supplement: Attachment — Submitted filename: Response to Reviewers.docx [file pone.0324056.s003.docx]

**ANSWER TO REVIEWER – REVIEWER 1**

Reviewer #1: my concerns have been duly worked on. I recommend for publication. In addition to other reviewers comments, I believe the authors duly made their writing and communication much clearer, wit an added emphasis on proper data visualization.

Thank you very much for your publication recommendation.

**ANSWER TO REVIEWER – REVIEWER 5**

Reviewer #5: The review is thorough, covering a broad range of oral targeted therapies for lung cancer in patients with hepatic and renal impairment.

Thank you very much for your review of this manuscript.

Table 1, which talks about summary of dose adjustments, is critical but lacks an interpretation section.

We have added the description on Table 1 as can be seen below.

Figure 3 could be referenced more explicitly in the discussion. (Instead of saying "Key findings are summarized in Fig. 3," specifying which insights the figure conveys makes more sense in my opinion.)

We have added the description on Figure 3 as can be seen below.

**ANSWER TO REVIEWER – REVIEWER 6**

Reviewer #6: Great work overall, with succinct analysis. I have a few comments that would further enhance the flow and impact of the article.

Thank you very much for your time and consideration to this manuscript.

1. In your work, you effectively categorized targeted therapies under various specific considerations, such as EGFR, ALK, BRAF/MEK, etc., and provided key conclusions for each therapy within these sections. To enhance the reader's comprehension, it would be immensely beneficial to include a brief explanation at the beginning of each section about these considerations. For example, a concise description of EGFR inhibitors (what they are and why they are important in targeted therapy) would provide essential context before delving into the analysis of specific therapies.

I sincerely value your concern and fully concur with this issue. An introduction paragraph has been incorporated for each kind of targeted therapy, as detailed below.

2. For Table 1, consider adding an additional column to include details on the inhibitory considerations (e.g., EGFR, ALK, etc.) for each therapy. While I understand that space limitations might make this challenging, it might be informative. This is a suggestion not a strict recommendation.

I appreciate your suggestions very greatly. We completely agree that adding the drug class here would make the table easier to understand. In accordance with editor recommendations that we should not include figures in tables; we have also removed every drug's chemical structure.

3. While one of the key challenges resulting to limited studies in the pharmacokinetic analysis of the drugs due to lack of available data from manufacturers was highlighted, your discussion would benefit from mentioning more key challenges for the limited studies. Additionally, providing recommendations for researchers and manufacturers on overcoming these challenges could enhance the practical value of your discussion. This would elevate your work beyond the already well-articulated dosage recommendations, giving it broader applicability and impact.

Thank you for your concern about this matter. We agree with you and have added our recommendation to enhance the publication for hepatic and renal impairment as can be seen below.

**ANSWER TO REVIEWER – REVIEWER 7**

Reviewer #7: 1. Merits

Comprehensive Scope

The manuscript systematically surveys pharmacokinetic (PK) studies for 21 oral targeted therapies in lung cancer patients with renal and hepatic impairment, spanning PBPK, PopPK, PK, and case‐series designs. This breadth provides clinicians with a one‐stop reference for dose‐adjustment guidance.

Rigorous Methodology

Adherence to PRISMA guidelines and PROSPERO registration (CRD42024518123) underscore the authors’ commitment to transparency and reproducibility. Dual‐reviewer screening, standardized data extraction, and the use of established risk‐of‐bias tools strengthen the review’s credibility.

Novel Contribution

To our knowledge, this is the first systematic review focusing exclusively on PK–based dose recommendations for targeted lung cancer therapies in patients with organ impairment. The 16 new and 8 updated dosing recommendations add tangible value beyond existing package-insert guidance.

Clinical Relevance

By juxtaposing label recommendations against empirical PK data—including in hemodialysis and peritoneal dialysis—this work has immediate applicability in oncology practice, aiding in precision dosing to maximize efficacy and safety.

Thank you for your consideration for this manuscript.

2. Aspects for Improvement

Search Strategy Transparency

Detail the full search strings in the main Methods (or move from Table S1 into a Supplement with explicit Boolean operators and date ranges).

Clarify database coverage (e.g., specify date of last search for Web of Science, Scopus, PubMed).

The systematic search is outlined in Table S1 and can be replicated by substituting the "drug name" with any drug listed in Table S2. The search result of each medicine for each database is also presented in Table S2. All searches were conducted on May 1, 2024.

Risk‐of‐Bias Synthesis

Summarize key findings of the bias assessment in the Results rather than relegating details to S4–S6 Tables. A brief paragraph quantifying how many studies were at low vs. moderate vs. high risk will help readers gauge confidence in the recommendations.

We have included the quantity of studies exhibiting bias for each study type. There is only one study for PBPK that presents a moderate risk of bias.

Results Presentation

Unify and simplify dosing tables. Currently, Table 1 and S7 appear to overlap. A consolidated main-text table with columns “Drug | Impairment Category | ΔAUC/Cmax | Dose Recommendation” would increase readability.

Table S7 serves to complement Table 1, since it encompasses additional pharmacokinetic parameters derived from all included studies. Consequently, we excluded the AUC/Cmax for Table 1 as this measurement is not as significant in comparison to the dosage itself. AUC/Cmax is only the pharmacokinetic parameter that assessed to rationalize the dose. We have included them in Table S7 and in the manuscript itself. We aim to maintain Table 1's compactness to ensure the report remains concise. We have amended Table 1 in accordance with Reviewer 6's suggestion by including the medication class category for each targeted therapy.

Include a PRISMA flow diagram as Figure 2 in the main text rather than only referencing it.

The PRISMA flow diagram serves solely as a guideline for authors in constructing a flow diagram, as evidenced by the information available on this website: https://www.prisma-statement.org/prisma-2020-flow-diagram. We reference the flow diagram on this website.

We have indeed created this type of figure on paper in accordance with the PRISMA statement as can be seen in Figure 2.

Discussion Depth & Limitations

Expand on clinical decision-making. For example, discuss how TDM or pharmacogenetic testing might integrate with these dosing recommendations in real‐world settings.

Thank you for your recommendation for this part. We have added a paragraph for current progress in pharmacogenetic and TDM. Hopefully, it will increase the discussion section.

Explicitly state limitations. The manuscript currently truncates before a standalone “Limitations” subsection. Address heterogeneity in study designs, missing data for certain eGFR or Child–Pugh classes, and language/publication bias.

In the prior version of this manuscript, we explicitly stated the limitations. The diverse study designs do not contribute to heterogeneity, as each study is undertaken for different objectives. There is no language bias as all studies are conducted in English. We assert that the limitations outlined in our manuscript have been sufficiently addressed.

Manuscript Organization & Clarity

Remove embedded “Response to Reviewers” text from the manuscript draft. That content belongs in your cover letter or tracked-changes response, not the submitted article itself.

We have confirmed that all manuscript components are arranged according to the journal's standards. We apologize if we made an error in the earlier communication.

Proofread for redundancy and English usage. A few sections (e.g., Reviewer #5 responses) are duplicated verbatim. Consolidate and streamline to avoid reader confusion.

We have ensured that our paper has been re-evaluated for grammar and word choice. If you believe there are specific words or sections in our document that exhibit duplication or redundancy, please provide us with the exact parts.

Figures & Visual Summary

Add a summary schematic (e.g., a heatmap or color-coded chart) showing which drugs require dose reduction by impairment severity.

We assert that the integration of Table 1 and Figure 3 sufficiently communicates the dose information to the doctor. Incorporating an additional summary schematic per your suggestion will result in repetition in the utilization of tables and figures within this work.

Ensure all figures (including the Biorender diagram) are high resolution and clearly labelled.

We confirmed that all figures are 300 dpi; nevertheless, the PDF version of this publication may exhibit low resolution due to the journal's automatic compression. I have included all the images here.
