# Supplementary material for: Tailoring oral targeted therapies dosage in lung cancer: A systematic review of pharmacokinetics studies on renal and hepatic impairment
Source: PLoS One. 2025 Jul 29;20(7):e0324056. doi: 10.1371/journal.pone.0324056 (PMC12306784; doi:10.1371/journal.pone.0324056)
Supplement: Attachment — Submitted filename: Response_to_Reviewers_auresp_2.docx [file pone.0324056.s005.docx]

**Comments to the Author**

**Reviewer #4: The authors presented their findings on "Tailoring Oral Targeted Therapies Dosage in Lung Cancer: A Systematic Review of Pharmacokinetics Studies on Renal and Hepatic Impairment." While the work is scientifically sound, there are opportunities for enhancement.**

**To improve the manuscript, I recommend that the authors provide more detailed explanations and discussions throughout the text. Clarifying the implications of the findings, particularly regarding how renal and hepatic impairments influence drug dosing, will enrich the overall narrative.**

I greatly appreciate your insightful review. We have reviewed our manuscript and revised several typographical errors regarding the influence of renal impairment on drug dosing. Hopefully, it increases the understanding of drug dosing in renal impairment.

We have added an explanation regarding how liver impairment can influence drug dosing, with the aim of enhancing readability and comprehension.

**Additionally, including more context on the relevance of pharmacokinetics in tailoring therapies for lung cancer patients would strengthen the manuscript's impact. Overall, a more thorough discussion will make the work more accessible and informative for readers.**

We have included information regarding the necessity of personalizing targeted therapy dosages in lung cancer, exemplified by erlotinib, one of the most prevalent medications for this condition. Low blood levels of erlotinib markedly reduced the objective response rate (ORR), whereas excessive dosages substantially heightened its toxicity.

Consequently, certain targeted therapies have been recommended for regular monitoring through therapeutic drug monitoring (TDM) to mitigate side effects and enhance efficacy, as elucidated in the subsequent paragraph.

We have also included a discussion on the significance of drug dosing in end-stage renal disease. It is hoped that this will enhance the significance of our systematic review in the future development of targeted therapy research.

**Reviewer #5: There are minor inconsistencies in your statistical analysis. Great work overall, but kindly ensure to include figures that explain your statistical analysis.**

We sincerely appreciate your feedback on our manuscript. We acknowledge that our systematic review in pharmacokinetic research methods may be unfamiliar. Thus, we present a graph to illustrate research methodologies, as depicted in Figure 1. This manuscript is a systematic review, not a meta-analysis; therefore, we do not conduct statistical analysis. Consequently, we are unable to provide figures that elucidate our statistical analysis.
