# Supplementary material for: Tailoring oral targeted therapies dosage in lung cancer: A systematic review of pharmacokinetics studies on renal and hepatic impairment
Source: PLoS One. 2025 Jul 29;20(7):e0324056. doi: 10.1371/journal.pone.0324056 (PMC12306784; doi:10.1371/journal.pone.0324056)
Supplement: Attachment — Submitted filename: Response to reviewer.docx [file pone.0324056.s007.docx]

**Comments to the Author**

**Reviewer #1:** There are minor inconsistencies in the body as well as some experiments or conclusions not explained. Great work overall, but kindly ensure you explain some of the results in a more details fashion.

For example, 'A PK study in HD patients showed no significant difference in AUC and Cmax'. Why do you think this happened? Would like a more detailed discussion on this.

This paragraph already contains an elucidation of this phenomenon. Initially, it appears that renal failure does not affect the elimination of erlotinib. Therefore, there is no change in the elimination of erlotinib in end stage renal disease patients, who are typically undergoing hemodialysis. Nevertheless, a study conducted on hemodialysis patients revealed a tendency towards a decrease in the area under the curve (AUC). This could be attributed to hypoalbuminemia, a condition commonly observed in patients undergoing hemodialysis, which leads to a decrease in the overall concentration of erlotinib in the body.

The explanation of hypoalbuminemia condition related to drug elimination also has been explained in discussion.

Work is also scientifically sound but can be made better when the explanations/discussions are clearly detailed out.

We have incorporated three key points into the discussion.

1. The FDA guidance on conducting drug studies for liver impairment and recommendations to manufacturers.

2. The FDA guidance on conducting drug studies for renal impairment and recommendations to manufacturers.

3. Factors to consider in patients with end-stage renal disease undergoing hemodialysis (HD) or continuous ambulatory peritoneal dialysis (CAPD)

Presumably, this addition has ability to enhance the comprehensiveness of our study. If there are any discussions that you believe should be included, please inform us.

Lastly, a minor observation is some abbreviations were not defined in the body of work. Fixing this would be great.

We have added HD, CAPD, Cl/F, ALP, CYP, p-gp, BCRP, AUC_ss_ and PRISMA abbreviation in the manuscript. We have assessed all manuscript and have put all abbreviation definition in the first of this abbreviation exist

**Reviewer #2:** Lung cancer, the foremost cause of cancer-related deaths globally, presents significant management challenges, especially in its advanced stage. This systematic review focuses on the dosage adjustments of targeted therapies for patients with concurrent renal and hepatic impairments, a frequent complication in cancer patients. By examining the package insert recommendations against the backdrop of available pharmacokinetic studies, the authors provide a comprehensive evaluation of the current practices. However, my only wish is that the authors had included a figure to summarize the work, which would have made it more appealing and accessible to the readers

I greatly appreciate your review. Figure 2 and its corresponding explanation have been included in the manuscript, as shown below. In addition, we have incorporated the utilization of Biorender as our software for creating this Figure in the method section.

**Reviewer #3:** The manuscript is well-prepared. However, I recommend the following revisions:

Expand the Methods section to provide more detailed information.

We greatly appreciate your review. We have reviewed the Methods section and believe that it is adequately detailed to allow reproducibility by other researchers. Additionally, we have included information about the use of resources such as PubChem and BioRender in our systematic review, which can be found in the manuscript. We also have included more comprehensive inclusion and exclusion criteria and why we used these criteria. If there are any specific aspects of the Methods section that you feel require further elaboration, please let us know, and we will gladly address them.

Ensure that the PRISMA guidelines are highlighted and adhered to within the Methods section.

After conducting a thorough review of the PRISMA guideline, we have ensured that all applicable items have been completed in our systematic review. The only exception is the statistical analysis, which can only be performed in a meta-analysis. The comprehensive information regarding our PRISMA guideline adherence can be found in File S1.

Including the chemical structure of each drug in the Results section could enhance readers' understanding.

We have added the chemical structure and PubChem CID of each drug in Table 1.
