# Supplementary material for: Tailoring oral targeted therapies dosage in lung cancer: A systematic review of pharmacokinetics studies on renal and hepatic impairment
Source: PLoS One. 2025 Jul 29;20(7):e0324056. doi: 10.1371/journal.pone.0324056 (PMC12306784; doi:10.1371/journal.pone.0324056)
Supplement: Attachment — Submitted filename: Answer to reviewer.docx [file pone.0324056.s008.docx]

**Journal office**

Thank you for submitting your revised manuscript. However, we note that our previous request "As your systematic review aims to inform clinical decision making, please include a certainty of evidence assessment (e.g.: GRADE) in your revised manuscript." has been overlooked.

We do note that you included a quality assessment, but  a certainty of evidence assessment is also required for your study. Quality assessment focuses on evaluating the internal validity of individual studies included in a review, looking for biases and limitations in their design, execution, and reporting. Certainty of evidence, on the other hand, refers to the overall confidence in the findings of the review, considering the quality of the included studies, but also the consistency of results, and other factors that might impact the reliability of the findings.

Given that your systematic review aims to inform clinical decision making, please also include a certainty of evidence assessment (e.g.: GRADE) in your revised manuscript.

I greatly appreciate your insight. We overlook that GRADE might be employed for narrative recommendations, rather than solely relying on RR or OR. We have included the methodology for conducting this GRADE, as illustrated below.

We also have added the level of certainty for each proposed new or revised recommendation in Table 1.

Furthermore, we added the description in the result section as can be seen below. All grade recommendation for new and updated recommendation can be seen in Table S8, S9, and S10

**Reviewer 7**

1. Search strategy transparency
The main text refers to “Comprehensive keyword usage” in Table S1 but does not detail full search strings.

Suggestion: Move critical elements of the search strategy (e.g., exact terms, date ranges) into the main Methods or an accessible appendix.

We appreciate your suggestion; it has been incorporated into Supplemetary Table 1 regarding the complete search string. As indicated in the methodology, we conducted a comprehensive search of the database on May 1, 2024. We hope that Table S1 has met your recommendations.

2. Handling of heterogeneity and narrative synthesis
While heterogeneity precludes meta‐analysis, the narrative discussion often lists findings without synthesizing overarching patterns.

Suggestion: Introduce summary tables or decision algorithms grouping drugs by degree of impairment (e.g., no adjustment vs. dose reduction vs. avoid) to guide clinicians.

I appreciate your recommendation regarding this issue. We recognize your analogous comments from your prior review process, in particular: “Add a summary schematic (e.g., a heatmap or color-coded chart) showing which drugs require dose reduction by impairment severity.”

Nonetheless, as previously said, including an additional summary table would be considered redundancy, as Table 1 have adequately conveyed the information, in our view. Moreover, it cannot be categorized according to the severity of impairment, as several studies employ differing classifications for renal and hepatic impairment. For example, Afatinib employed an eGFR threshold of <15, whereas dacomitinib utilized <30, and brigatinib adopted <10, as illustrated below.

Upon reevaluation of Table 1, it effectively elucidates this issue. Clinicians would utilize this type of table more efficiently, as they typically reference guidelines indicating which drug should be administered and check this table to modify the dosage if their patients have hepatic or renal impairment. We cannot draw the "avoid" recommendation as no study has indicated such. Therefore, this recommendation is not clinically informative when based on the degree of hepatic or renal failure.

3. Limitations section
A concise, dedicated Limitations paragraph is missing. Key issues include: publication bias toward positive PK results, reliance on small case series for dialysis data, and variability in impairment classification (Child–Pugh vs. NCIc).

Suggestion: Explicitly acknowledge these and discuss their impact on generalizability.

In the prior revision, we have explicitly incorporated the study's limitations.

In this revision, we have incorporated your recommendation concerning the limitations of this study as can be seen below.

4. Evidence gaps and unsupported statements
Several package‐insert claims (e.g., for vemurafenib, entrectinib, selpercatinib) lack citable PK publications.

Suggestion: Either provide citations or rephrase these as “manufacturer‐reported” and flag the need for independent confirmation.

I greatly appreciate your recommendations. We agree that this comment, solely reported by the manufacturer, should be emphasized. Consequently, we have included a "c" remark in Table 1 for vemurafenib, larotrectinib, entrectinib, capmatinib, selpercatinib, pralsetinib, and sotorasib, as illustrated below.

We also have cited the website where we acquired the drug label/package insert.

5. Clarity and conciseness of Discussion
The Discussion is lengthy and occasionally repetitive, particularly in describing pathophysiology.

Suggestion: Streamline by focusing on the most clinically actionable points and moving detailed mechanistic explanations to a separate subsection or supplemental material.

Thank you for your suggestions. We have deleted several parts regarding basic pharmacokinetic of the drug as can be seen below.

6. Formatting and adherence to journal guidelines
Financial disclosure and ethics statements appear as placeholders in the submission form but are not integrated into the manuscript text.

Suggestion: Ensure that all PLOS ONE submission requirements (funding sources, competing interests, data availability) are clearly stated in the manuscript.

The manuscript has included conflict of interest, funding sources, and data availability. If any phrasing need alteration, it can be addressed by the editor or typesetter.

7. Consistency in dosing recommendations
In some sections, you propose new recommendations that differ subtly from package insert guidance without clear rationale.

Suggestion: For each proposed change, briefly summarize the magnitude of PK alterations (e.g., “AUC↑50% in CP C, therefore reduce dose by 50%”) to justify the recommendation.

We observed that you repeated these similar comments in the prior review process. We believe we have addressed these comments in the prior review round, except for afatinib. As demonstrated here, afatinib has significant variability in AUC for patients with an eGFR of 15-29, indicating that its dosage may not be reduced as aggressively as other targeted therapies. Consequently, we concur with the medication label's recommendation to reduce it by 25%. We have included all AUCs referenced in the result section of each drug.

Afatinib

Alectinib

Brigatinib

Crizotinib

Lorlatinib

Trametinib

Larotrectinib

Selpercatinib
